# Cyp6g2 is the major P450 epoxidase responsible for juvenile hormone biosynthesis in *Drosophila melanogaster*

**DOI:** 10.1186/s12915-024-01910-4

**Published:** 2024-05-13

**Authors:** Qiangqiang Jia, Liu Yang, Jiamin Wen, Suning Liu, Di Wen, Wei Luo, Weihua Wang, Subba Reddy Palli, Li Sheng

**Affiliations:** 1 Guangdong Provincial Key Laboratory of Insect Developmental Biology and Applied Technology, Institute of Insect Science and Technology & School of Life Sciences, South China Normal University, Guangzhou, China; 2 Guangmeiyuan R&D Center, Guangdong Provincial Key Laboratory of Insect Developmental Biology and Applied Technology, South China Normal University, Meizhou, China; 3 College of Biological Science and Agriculture, Qiannan Normal University for Nationalities, Duyuan, 558000 China; 4 Center of Pharmaceutical Technology, Tsinghua University, Beijing, 100084 China; 5 Department of Entomology, College of Agriculture, Food and Environment, University of Kentucky, Lexington, KY 40546 USA

**Keywords:** Juvenile hormone (JH), Farnesoic acid (FA), Epoxidase, CYP15, Cyp6g2

## Abstract

**Background:**

Juvenile hormones (JH) play crucial role in regulating development and reproduction in insects. The most common form of JH is JH III, derived from MF through epoxidation by CYP15 enzymes. However, in the higher dipterans, such as the fruitfly, *Drosophila melanogaster*, a bis-epoxide form of JHB3, accounted most of the JH detected. Moreover, these higher dipterans have lost the *CYP15* gene from their genomes. As a result, the identity of the P450 epoxidase in the JH biosynthesis pathway in higher dipterans remains unknown.

**Results:**

In this study, we show that Cyp6g2 serves as the major JH epoxidase responsible for the biosynthesis of JHB3 and JH III in *D. melanogaster*. The *Cyp6g2* is predominantly expressed in the *corpus allatum* (CA), concurring with the expression pattern of *jhamt*, another well-studied gene that is crucial in the last steps of JH biosynthesis. Mutation in *Cyp6g2* leads to severe disruptions in larval-pupal metamorphosis and exhibits reproductive deficiencies, exceeding those seen in *jhamt* mutants. Notably, *Cyp6g2*^*−/−*^*::jhamt*^*2*^ double mutants all died at the pupal stage but could be rescued through the topical application of JH analogs. JH titer analyses revealed that both *Cyp6g2*^*−/−*^ mutant and *jhamt*^*2*^ mutant lacking JHB3 and JH III, while overexpression of *Cyp6g2* or *jhamt* caused a significant increase in JHB3 and JH III titer.

**Conclusions:**

These findings collectively established that Cyp6g2 as the major JH epoxidase in the higher dipterans and laid the groundwork for the further understanding of JH biosynthesis. Moreover, these findings pave the way for developing specific Cyp6g2 inhibitors as insect growth regulators or insecticides.

**Supplementary Information:**

The online version contains supplementary material available at 10.1186/s12915-024-01910-4.

## Background

Juvenile hormones (JHs) are vital in controlling insect development, metamorphosis, and reproduction. In larval/nymphal phase, JH exerts status quo function and prevents precocious metamorphosis by suppressing the molting hormone 20-hydroxyecdyonse (20E) signaling. In the adult stage, JH promotes many aspects of reproduction including previtellogenic development, vitellogenesis, oogenesis, and ovulation [1, 2]. They are a group of structurally related acyclic sesquiterpenoids produced by the *corpora allata* (CA), a pair of specialized minute endocrine glands located adjacent to the brain. To date, seven forms of JHs have been identified, distinguished by variations in side-chain length or epoxidation status, that is, JH0, JH I, 4-methyl JH I, JH II, JH III, JH III bisepoxide (JHB3), and JH III skipped bisepoxide (JHSB3) [3, 4]. JH III is the predominant or sole JH compound found in most insects. JHB3 was discovered in higher dipterans like the fruitfly, *Drosophila melanogaster*. JHSB3 was detected in hemipterans. Other types of JH were identified in lepidopterans. Methyl farnesoate (MF) serves as the direct precursor of JH III, as well as JHB3 in the *D. melanogaster*, and also exhibits moderate JH activity in certain insects [5]. JH homologs exert their effects through the intracellular JH receptor Methoprene-tolerant (Met), a ligand-activated bHLH-PAS transcription factor. Upon JH exposure, Met forms complexes with another bHLH-PAS protein called Taiman (Tai), and together they regulate the transcription of downstream response genes, such as *Krüppel-homolog 1* (*Kr-h1*) [6–9]. Additionally, JH could exert rapid non-genomic effects through membrane signaling [10, 11], particularly in the opening of intercellular channels in the follicular epithelium for vitellogenin (Vg) transport into the oocytes [12, 13].

The biosynthesis of JH is conventionally divided into the early steps (mevalonic acid pathway, MVAP) and the late steps (JH-branch pathway). In the early steps, farnesyl pyrophosphate (FPP) is synthesized through the MVAP. Subsequently, in the late steps, FPP is hydrolyzed to farnesol, which is then oxidized to farnesal and farnesoic acid (FA). FA is finally converted to JH III through two terminal reactions: methylation by a juvenile hormone acid methyltransferase (JHAMT) and epoxidation by a P450 epoxidase. The order of these two terminal steps varies depending on the insect orders. In most insects, JHAMT converts FA into MF, and then P450 epoxidase (MF epoxidase CYP15A1) epoxidates MF into JH III. However, in Lepidoptera, methylation occurs after epoxidation. JH I/II/III acids (JHAs) are initially generated by P450 epoxidase (farnesoic acid epoxidase CYP15C1), and then they are methylated to yield the corresponding active JH compounds [14–17]. Additionally, in *T. castaneum*, CYP15A1 acts as a mixed farnesoic acid/MF epoxidase [18]. The key enzyme CYP15 but lacking in higher dipterans, involved in sesquiterpenoid epoxidation, represents a major evolutionary novelty among insects and significantly impacts reproductive fitness [19]. Because the late steps enzymes in JH biosynthetic pathway are highly specific to insects, they have long been regarded as promising targets for discovering novel insecticides [4].

Decades ago, it was discovered that the CA of *D. melanogaster*, a well-studied organism in biological research, produces and releases three JHs: JHB3, JH III, and MF [20]. Previous studies have suggested that JHB3 is synthesized from FA through initial epoxidation and terminal methylation [21, 22]. However, investigations on JH epoxidase in *D. melanogaster* have lagged far behind those in other insects due to the absence of the CYP15 ortholog in higher Diptera (also called Cyclorrhapha), which includes *D. melanogaster*. Consequently, the specific identity of the JH epoxidase in higher Diptera remains unknown. The protein most closely related to CYP15 in *D. melanogaster* is Cyp305a1 and Cyp303a1 [4, 19]. The function of Cyp305a1 is still unknown, although its orthologs are found as a single gene in most Neoptera species [19]. While Cyp303a1 has been identified as having conserved regulatory function in adult eclosion and wing expansion in *D. melanogaster* and *L. migratoria*, its endogenous substrate is still not identified [23, 24]. Another promising candidate is Cyp6g2, a P450 enzyme that is highly expressed in the CA of *D. melanogaster*. Global RNAi knockdown of *Cyp6g2* results in pupal lethality [25, 26]. However, several studies have shown its involvement in detoxifying insecticides [27–29], leaving its precise biochemical function and the definitive role in JH biosynthesis unknown [23]. In this study, we have successfully proved that Cyp6g2 serves as the major JH epoxidase in *D. melanogaster*. As the most prominent model insects, characterization of JH epoxidase in *D. melanogaster* will promote research in the JH field and evolution of higher Dipterans. These studies would provide important information that could help establish the foundation for developing new approaches for the control of harmful higher dipterans.

## Results

### The spatiotemporal expression pattern of Cyp6g2 is similar to jhamt

In *D. melanogaster*, JH is known to play vital roles in post embryonic development and reproduction [30, 31]. To examine whether *Cyp303a1*, *Cyp305a1*, and *Cyp6g2* have any function in the CA, we performed CA-specific knockdown of *Cyp305a1*, *Cyp303a1*, and *Cyp6g2* with *jhamt-Gal4* and examined phenotypes associated with JH deficiency [5]. Remarkably, only the knockdown of *Cyp6g2* resulted in partial pupal lethality, accompanied by decreases in adult ovary size and fecundity (Additional file 1: Fig. S1), suggesting that Cyp6g2’s involvement in JH biosynthesis. Therefore, we focused on Cyp6g2 in subsequent studies.

**Fig. 1:**
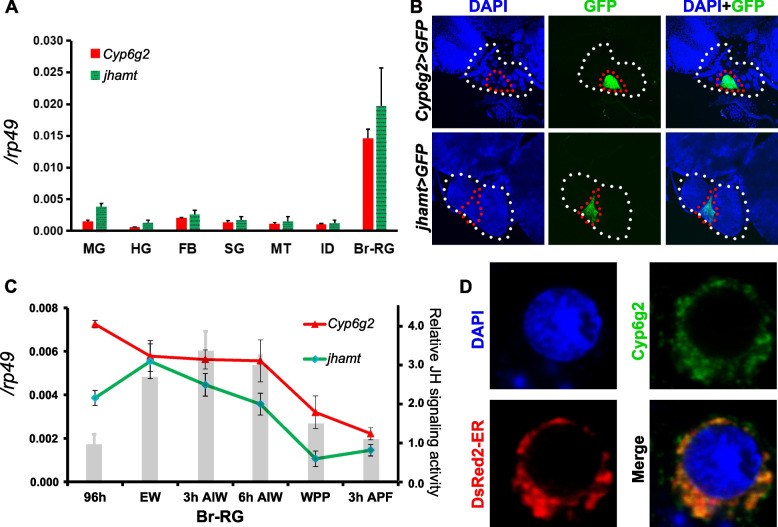
Spatiotemporal expression pattern of *Cyp6g2* and *jhamt*. **A** qRT-PCR measurements of tissue expression profile of *Cyp6g2* and *jhamt* in 3 h AIW larvae (*n* = 3). MG, midgut; HG, hindgut; FB, fat body; SG, salivary glands; MT, Malpighian tubules; ID, imaginal discs; Br-RG, brain-ring gland. **B** Images of the Br-RG complex in *Cyp6g2-GAL4* > *UAS-GFP* and *jhamt-GAL4* > *UAS-GFP* animals. GFP was specifically located in the CA. **C** qRT-PCR analysis showing temporal expression of *Cyp6g2* and *jhamt* in Br-RG complex (left axis) (*n* = 3), and changes in the JH signaling activity revealed by relative LacZ fluorescence intensity in the fat body of *JHRR-LacZ* (right axis), modified from our previously published results [33]. AIW, after initiation of wandering stage; EW, early wandering stage; WPP, white prepual stage; APF, after puparium formation. **D** Subcellular localization of Cyp6g2. The confocal section of Kc cells transfected with V5-tagged Cyp6g2 (green) and DsRed2-ER (red)

Previous studies have consistently shown that the expression of *jhamt* is predominantly localized in the CA, exhibiting a spatial and temporal overlap with JH biosynthesis [16, 17, 32]. We used qRT-PCR to analyze the tissue specificity of *Cyp6g2* expression, with *jhamt* as a positive control. Similar to *jhamt*, *Cyp6g2* transcripts were predominantly detected in the brain-ring gland (Br-RG) complex of larvae at 3 h after the initiation of wandering (3 h AIW) stage (Fig. 1A). To further explore the spatial localization of *Cyp6g2*, *Cyp6g2-Gal4* transgenic flies were established. After crossing with *UAS-GFP* flies, the fluorescence signal was located specifically in the larval CA cells but not in the prothoracic gland or corpora cardiac cells of the RG, just like *jhamt-Gal4* flies (Fig. 1B). Subsequently, we examined the changes in *Cyp6g2* and *jhamt* mRNA levels in the Br-RG complex during early 3rd instar to 3 h after white prepupal formation stage (3 h APF). Elevated *Cyp6g2* and *jhamt* transcript levels were observed during the feeding stage (96 h after egg laying) and the wandering stage, corresponding to the glands’ maximal JH biosynthetic activity. Conversely, mRNA levels became low during white prepupal stage and afterwards, when JH biosynthesis is low (Fig. 1C). Furthermore, the developmental profile of *Cyp6g2* and *jhamt* closely mirrored changes in JH signaling activity reflected by the fluorescence intensity of *JHRR-LacZ* reporter as we previously described [33] (Fig. 1C). To explore the subcellular localization of Cyp6g2, V5 epitope-tagged Cyp6g2 were co-transfected into Kc cells with a DsRed2-tagged marker for the endoplasmic reticulum or mitochondria. The result showed that Cyp6g2 is co-localized with the endoplasmic reticulum marker (Fig. 1D), in agreement with the previous observations that JH epoxidases are usually localized in the endoplasmic reticulum [15]. Collectively, these results underscore a similar expression pattern between *Cyp6g2* and *jhamt*, further substantiating the involvement of *Cyp6g2* in JH biosynthesis.

### Developmental defects of Cyp6g2 mutant

**Fig. 2:**
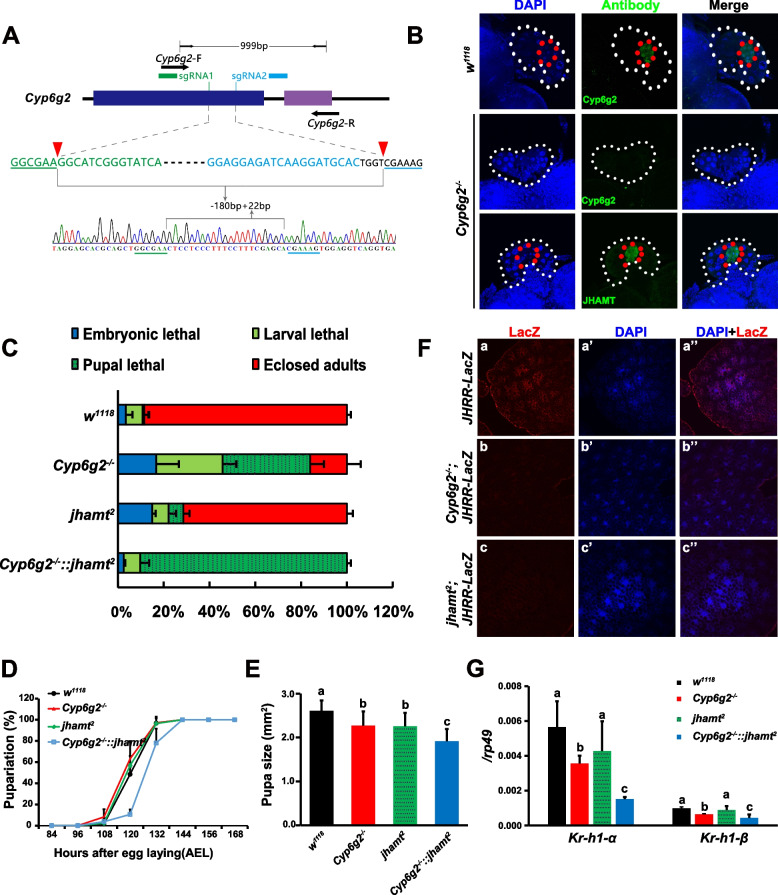
Generation of a CRISPR/Cas9 mediated *Cyp6g2* mutant and analysis of developmental phenotypes. **A** Shematic diagram of the CRISPR/Cas9-mediated knockout of *Cyp6g2*, showing the two exons of *Cyp6g2* in *D. melanogaster* genome, the positions and target sequences of two sgRNAs and a representative chromatogram. **B** Immunohistochemistry to detect Cyp6g2 in the CA of *w*^*1118*^ and *Cyp6g2*^*−/−*^ mutants, and JHAMT in the CA of *Cyp6g2*^*−/−*^ mutants at 3 h AIW. **C** The lethality of *w*^*1118*^, *Cyp6g*^*−/−*^, *jhamt*^*2*^, and *Cyp6g2*^*−/−*^*::jhamt*^*2*^ homozygous mutants during embryonic, larval, and pupal stages (*n* = 3). **D** Pupariation timing of the abovementioned four genotype animals (*n* = 3). **E** Pupa size of the abovementioned four genotype animals (*n* = 20). **F** Comparison of LacZ levels (red) in the fat body of *JHRR-LacZ* under the genetic background of *w*^*1118*^, *Cyp6g*^*−/−*^, and *jhamt*.^*2*^. Cell nuclei were labeled with DAPI (blue). **G** Expression changes of *Kr-h1-α* and *Kr-h1-β* in the whole body of the abovementioned four genotype animals (*n* = 3)

We thus constructed *Cyp6g2* mutants by employing a dual sgRNA-directed CRISPR-Cas9 system. A *Cyp6g2*^*−/−*^ mutant line with a 180-bp sequence-deleted and an additional 22-bp nucleotide insert was obtained (Fig. 2A). The JHAMT antibody was previously generated in our lab [9], and we here generated the Cyp6g2 antibody. The immunofluorescence staining confirmed the absence of Cyp6g2 protein and the presence of JHAMT protein in the CA of the *Cyp6g2*^*−/−*^ mutant. Compared to *w*^*1118*^ animals, *Cyp6g2*^*−/−*^ and *jhamt*^*2*^ mutants exhibited higher embryonic and pupal lethality. However, the larval and pupal lethality of *Cyp6g2*^*−/−*^ mutants reached to 29.0 and 28.1% respectively, being much higher than that of *jhamt*^*2*^ mutants (7.1 and 6.5% respectively). Approximately 26.1% of *Cyp6g2*^*−/−*^ embryos survived to adulthood whereas 71.4% *jhamt*^*2*^ embryos did (Fig. 2C). To better understand the relationship between *Cyp6g2* and *jhamt* in the biosynthesis of JHs, *Cyp6g2*^*−/−*^*:: jhamt2* double mutants were generated. Interestingly, the embryonic and larval lethality of the double mutants was lower than that of single *Cyp6g2* or *jhamt* mutant, but the double mutants typically died during the pupal stage (Fig. 2C). Pupariation time was not delayed in *Cyp6g2*^*−/−*^ and *jhamt*^*2*^ single mutants, but was delayed about 12 h in *Cyp6g2*^*−/−*^*:: jhamt2* double mutants (Fig. 2D). The pupae of *Cyp6g2*^*−/−*^ or *jhamt*^*2*^ was smaller than that of *w*^*1118*^, and even smaller in *Cyp6g2*^*−/−*^*:: jhamt*^*2*^ double mutants (Fig. 2E). The delay in pupariation time and reduction in pupal size was similar to those observed in previously reported allatectomized animals (*Aug21* > *grim*) and *Met*^*27*^*:: Gce*^*2.5 k*^ double mutants [8, 34, 35]. Using *JHRR-LacZ* as a JH signaling activity indicator, we observed a significant decrease in JH signaling in the fat body of *Cyp6g2*^*−/−*^ and *jhamt*^*2*^ single mutants at 3 h AIW (Fig. 2F). *Kr-h1* is a crucial JH primary response gene and acts as a repressor of insect metamorphosis [36]. At 3 h AIW, the whole body expression levels of *Kr-h1-α* and *Kr-h1-β* were reduced significantly in *Cyp6g2*^*−/−*^ mutants and *Cyp6g2*^*−/−*^*:: jhamt2* double mutants, but not in the *jhamt*^*2*^ mutant (Fig. 2G). The composite studies reveal that Cyp6g2 is involved in JH biosynthesis and important for regulating development and metamorphosis.

### Female reproduction deficiencies in Cyp6g2 mutant

**Fig. 3:**
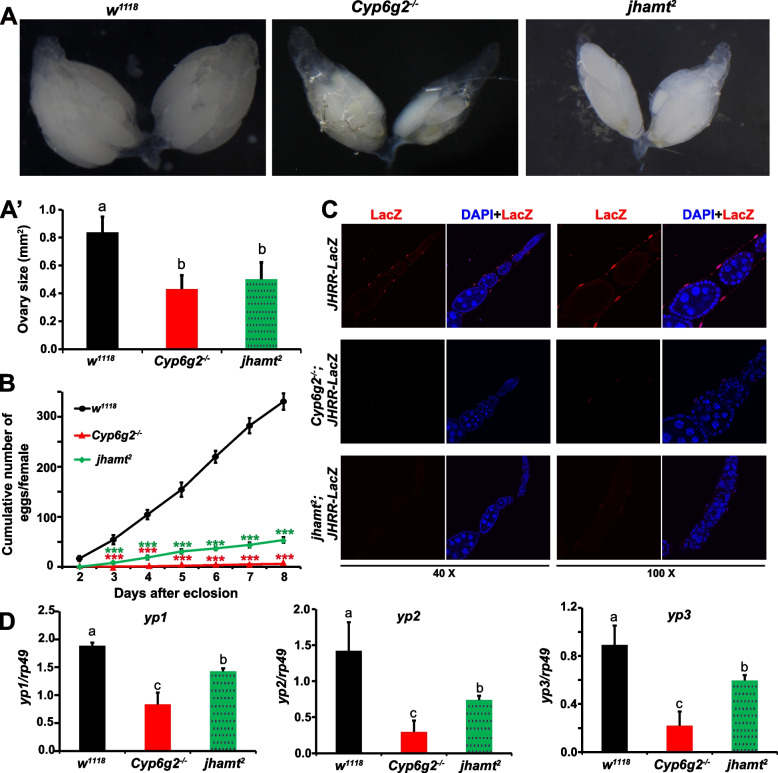
The female reproductive defects in the *Cyp6g2*^*−/−*^ mutant. **A,A'** The representative images of ovaries from *w*^*1118*^, *Cyp6g2*^*−/−*^ mutant and *jhamt*^*2*^ mutant in 6-day-old virgins. **A'** Quantification of ovary sizes shown in **A**, the bars labeled with different lowercase letters are significantly different (*p* < 0.05) (*n* = 10–15). **B** The cumulative number of eggs laid by per female in *w*^*1118*^, *Cyp6g2*^*−/−*^ mutant, and *jhamt*^*2*^ mutant (*n* = 6). **C** Comparison of LacZ levels (red) in the ovary of *JHRR-LacZ* under the genetic background of *w*^*1118*^, *Cyp6g*^*−/−*^, and *jhamt*^*2*^. Cell nuclei were labeled with DAPI (blue). **D** Quantification of mRNA of *yolk protein 1* (*yp1*), *yolk protein 2* (*yp2*), and *yolk protein 3* (*yp3*) in *w*^*1118*^, *Cyp6g2*^*−/−*^ mutant, and *jhamt*.^*2*^ mutant females by qRT-PCR (*n* = 4)

We have previously shown that artificial reduction of JH levels in *D. melanogaster* females led to a decrease in fecundity, manifested as reduced oviposition (egg laying) and smaller ovary size, by the partially genetic ablation of the CA or mutation of *jhamt* [31]. In this study, we found that the ovary size of the 7-day-old *Cyp6g2*^*−/−*^ virgin females was significantly reduced (Fig. 3A–A'), even smaller than that of *jhamt*^*2*^, notwithstanding the difference was not statistically significant. An observation on the fecundity of *w*^*1118*^, *Cyp6g2*^*−/−*^, and *jhamt*^*2*^ showed that mutation of *Cyp6g2* dramatically reduced the number of eggs laid by females. The cumulative oviposition number per female in *w*^*1118*^ was 330.3 ± 16.4 eggs at 8 days after eclosion, whereas this number decreased to 54 ± 5.8 eggs in *jhamt*^*2*^ and further plummeted to 6.0 ± 0.6 eggs in *Cyp6g2*^*−/−*^ (Fig. 3B). Consequently, the homozygous *Cyp6g2*^*−/−*^ mutant line could not be successfully maintained. Our previous study showed that JH signaling is mainly activated in ovarian muscle cells to promote ovulation [31]. As revealed by *JHRR-LacZ*, JH signaling was also significantly reduced in the ovarian muscle cells of *Cyp6g2*^*−/−*^ and *jhamt*^*2*^ (Fig. 3C).

JH plays an important role in stimulating yolk protein synthesis in the fat body to facilitate mature egg production [37]. Consistently, the mRNA levels of *yolk protein 1* (*yp1*), *yolk protein 2* (*yp2*), and *yolk protein 3* (*yp3*) were found to be downregulated in females lacking *Cyp6g2* and *jhamt* function (Fig. 3D). The composite data show that Cyp6g2 is indispensable for female reproduction.

### Rescue experiments by topical application of JH and its analogs

**Fig. 4:**
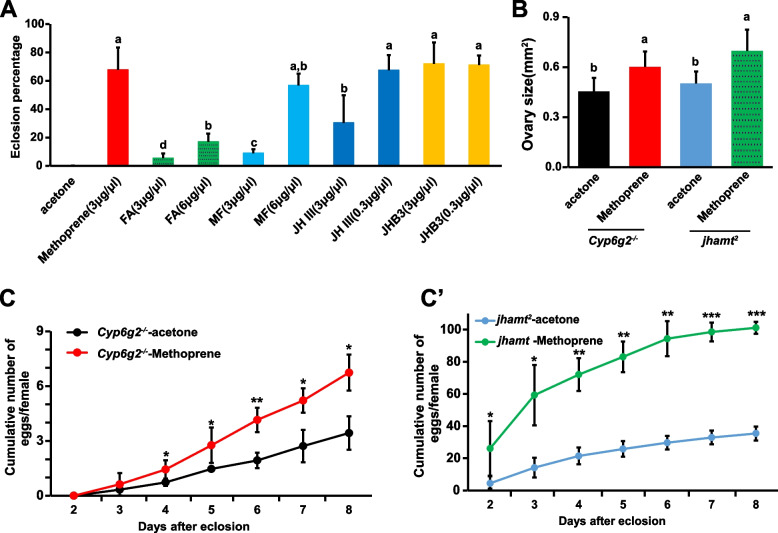
The rescue effects of JH or JH analogs to *Cyp6g2*^*−/−*^*::jhamt*^*2*^ double mutants and *Cyp6g2*^*−/−*^ single mutants. **A** The percentage of successfully eclosed adults by topical application of different doses of JH or JH analog to L3D1 larvae (*n* = 3). **B** The increase in ovary size by topical application of JH analog Methoprene to *Cyp6g2*^*−/−*^ and *jhamt*^*2*^ females on the day of eclosion (*n* = 10–15). **C–C'** The increase in egg production by topical application of JH analog Methoprene to *Cyp6g2*^*−/−*^ (**C**) and *jhamt*.^*2*^ (**C'**) females on the day of eclosion (*n* = 3)

The developmental lethality and reduced fertility caused by JH deficiency could be alleviated by the application of JH analog Methoprene either topically or in the diet [7, 8, 35]. In line with previous results, topical application of 0.5 μl of 3 μg/μl Methoprene to *Cyp6g2*^*−/−*^*::jhamt*^*2*^ on the L3D1 rescued 67.8% of animals to adults. Similarly, the same dose or a lower dose (0.3 μg/μl, 0.5 μl) of JHB3 exhibited a similar rescue effect, with 71.9 and 71.0% rescue efficiencies, respectively. However, when using JH III, the lower dose (0.3 μg/μl, 0.5 μl) showed a rescue efficiency of 67.5%, while the same dose (3 μg/μl, 0.5 μl) led to deterioration in the rescue effect (30.4%). In the case of MF, the low dose group (3 μg/μl, 0.5 μl) showed a rescue efficiency of 9%, whereas the high dose group (6 μg/μl, 0.5 μl) exhibited a similar efficiency (56%). Notably, FA also showed certain rescue effects, with a rescue efficiency of 5.4% in the low dose group (3 μg/μl, 0.5 μl) and 16.9% in the high dose group (6 μg/μl, 0.5 μl) (Fig. 4A). Likewise, the topical application of the Methoprene (3 μg/μl, 0.5 μl) partially restored ovary size and fecundity in the *Cyp6g2*^*−/−*^ mutant and *jhamt*^*2*^ mutant (Fig. 4B–C'). The rescue experiments confirmed the biosynthetic roles of Cyp6g2 in JH biosynthesis pathway and the physiological functions of Cyp6g2 in regulating development, metamorphosis, and reproduction.

### *Sesquiterpenoid titers in Cyp6g2*^*−/−*^* and jhamt*^*2*^* mutants*

**Fig. 5:**
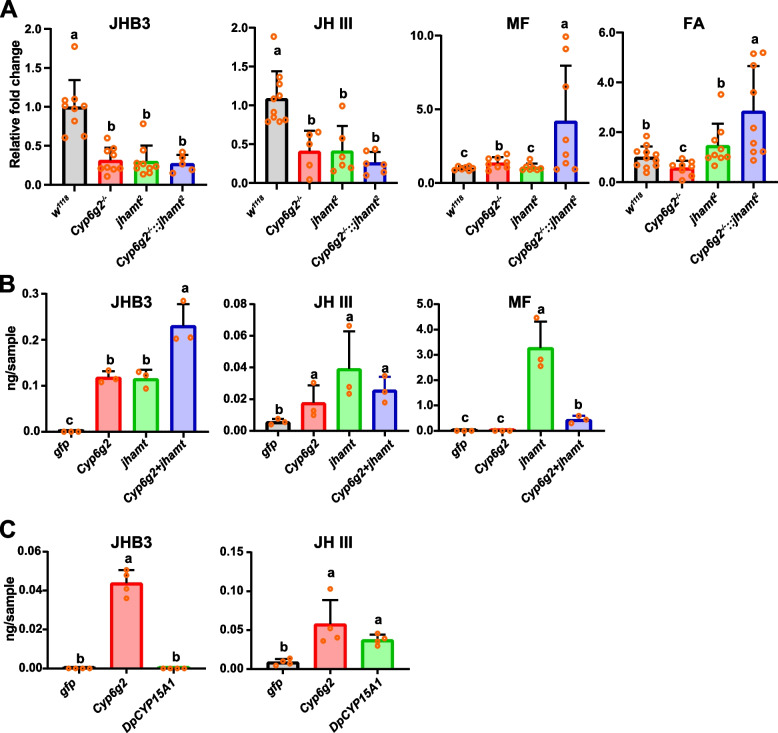
The analyses of sesquiterpenoid titers. **A** The changes of whole body sesquiterpenoid titers in the larvae of *w*^*1118*^, *Cyp6g*^*−/−*^, *jhamt*^*2*^, and *Cyp6g2*^*−/−*^*::jhamt*.^*2*^ homozygous mutants (*n* = 5–11). **B** The changes of JHB3, JH III, and MF titers in the Kc cells upon *Cyp6g2* overexpressed, *jhamt* overexpressed, and *Cyp6g2* and *jhamt* co-overexpressed. FA was supplemented as substrate (*n* = 3). **C** The changes of JHB3 and JH III titers in the Kc cells upon *Cyp6g2* or *DpCYP15A1* overexpressed. MF was supplemented as substrate (*n* = 4)

To better understand the relationship between the JH-deficient lethal phenotypes and the biosynthesis of the four sesquiterpenoids (JHB3, JH III, MF, and FA) by the larval CA, we measured the titers of these four sesquiterpenoids in the larval whole bodies of the *w*^*1118*^, *Cyp6g2*^*−/−*^, *jhamt*^*2*^, *Cyp6g2*^*−/−*^*::jhamt*^*2*^ at 3 h AIW. In comparison to the *w*^*1118*^ larvae, the JHB3 titer decreased by 70% in *Cyp6g2*^*−/−*^, *jhamt*^*2*^ and *Cyp6g2*^*−/−*^*::jhamt*^*2*^ larvae (Fig. 5A–JHB3). JH III titer decreased by 60% in *Cyp6g2*^*−/−*^ and *jhamt*^*2*^ larvae and further decreased by 70% in *Cyp6g2*^*−/−*^*::jhamt*^*2*^ larvae. However, no significant difference was observed between the double mutant group and the single mutant group in terms of JH III titer (Fig. 5A–JH III). The MF titer remained unchanged in *jhamt*^*2*^ larvae, but increased by 37% in *Cyp6g2*^*−/−*^ larvae, and further increased by three-fold in *Cyp6g2*^*−/−*^*::jhamt*^*2*^ larvae (Fig. 5A–MF). The FA titer also remained unchanged in *jhamt*^*2*^ larvae, but decreased by 43% in *Cyp6g2*^*−/−*^ larvae, and increased by 185% in *Cyp6g2*^*−/−*^*::jhamt*^*2*^ larvae (Fig. 5A–FA). These results provided direct evidence that Cyp6g2 participated in JHB3 and JH III biosynthesis, likely by using both FA and MF as substrates.

### Enzymatic activity of Cyp6g2 against FA and MF

**Fig. 6:**
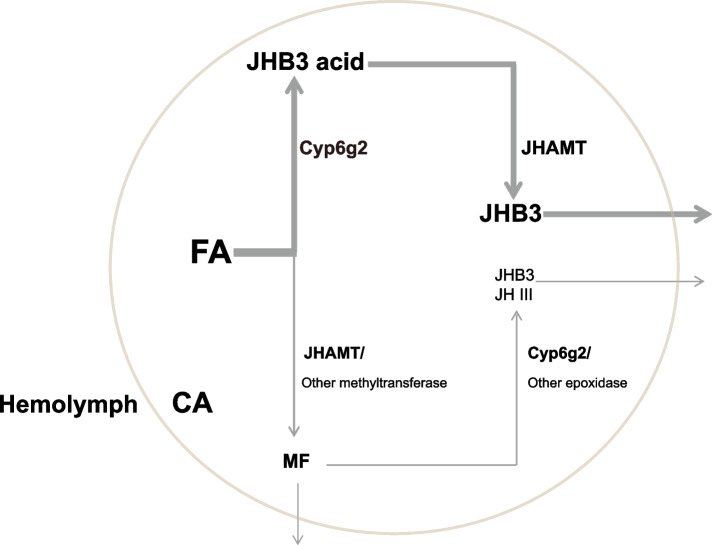
The schematics showing the last two steps of JH biosynthesis in *D. melanogaster.* In the CA, the vast majority of FA is epoxidized into JHB3 acid by Cyp6g2 firstly, and then methylated into JHB3 by JHAMT. A small proportion of FA is methylated into MF by JHAMT or other unknown methyltransferase firstly, then epoxidized into JHB3 or JH III by Cyp6g2 or other unknown epoxidase

To further clarify the roles of Cyp6g2 in the JH biosynthesis pathway, we examined the enzymatic activity of Cyp6g2 against two plausible substrates, FA and MF. We employed a transient expression system using *D. melanogaster* Kc cells. When *Cyp6g2* or *jhamt* was overexpressed individually and FA was added as a substrate, the production of JHB3 and JH III was significantly increased (Fig. 5B–JHB3, JH III). And the production of JHB3 was doubled upon co-overexpression of *Cypg2* and *jhamt* (Fig. 5B–JHB3). The production of MF was obvious when *jhamt* was overexpressed individually, but was markedly reduced upon co-overexpression of *Cyp6g2* and *jhamt* (Fig. 5B–MF). These results suggest that FA is mainly epoxidized into JHB3 acid by Cyp6g2, and next JHB3 acid was methylated into JHB3 by JHAMT; however, FA can be also minorly methylated into MF. We then tested whether Cyp6g2 could epoxidize MF, using DpCYP15A1 (*Diploptera punctate* CYP15A1) as a positive control. When *Cyp6g2* or *DpCYP15A1* was overexpressed and MF was added as a substrate, JHB3 was detected only in *Cyp6g2*-overexpressed group, but JH III was detected in both groups (Fig. 5C). These results showed that Cyp6g2 could epoxidize MF into JHB3 and JH III respectively. It is noteworthy that when FA was taken as a substrate, more JHB3 were produced (Fig. 5B, C). This showed that Cyp6g2 could not utilize MF efficiently. Taken together, we demonstrate that Cyp6g2 is the major P450 epoxidase in the JH biosynthesis pathway, using FA as the main substrate and MF as the minor substrate. Besides Cyp6g2 and JHAMT, we assume that other epoxidases and methyltransfereases are also involved in JH biosynthesis (Fig. 6).

## Discussion

### Cyp6g2 is the major P450 epoxidase responsible for JH biosynthesis in D. melanogaster

Significant advancements have been made since the original discovery that JHB3 as the predominant JH in *D. melanogaster* [20]. These include the identification of the JH receptor Met and Gce as well as the JH primary response gene *Kr-h1*, and the characterization of the molecular mechanism underlying cooperative regulation of JH and 20E on molting and metamorphosis [6–8, 20, 33, 34]. However, due to the universal JH epoxidase gene *CYP15* is not present in the genome of *D. melanogaster* and its congeners, the identity of JHB3 epoxidase remains elusive [23, 38]. Based on the following criteria, we propose that Cyp6g2 is the major epoxidase responsible for JH biosynthesis in *D. melanogaster*. First, JH is known to be present in the early larval instars, declines substantially during the last (third) larval stage, and then reappears transiently during pupariation in *D. melanogaster* [35]. Acting like *jhamt*, *Cyp6g2* is expressed selectively in the CA and its temporal expression pattern is closely correlated with JH signaling activity (Fig. 1) [16, 25, 33]. This parallels the expression profile of the first-described JH epoxidase gene *DpCYP15A1* (*Diploptera punctate CYP15A1*) [15]. Furthermore, Cyp6g2, like DpCYP15A1, also localized to the endoplasmic reticulum (Fig. 1D) [15]. Second, *Cyp6g2*^*−/−*^ mutants displayed JH-dependent lethality and female reproductive deficiencies (Figs. 2 and 3), and pupal lethality of *Cyp6g2*^*−/−*^*::jhamt*^*2*^ double mutants is 100%, identical to that of previously reported genetically allatectomized animals (*Aug21* > *grim*). Third, applying exogenous JH and JH analogs could rescue *Cyp6g2*^*−/−*^*::jhamt*^*2*^, allowing them to develop into adults. Furthermore, these treatments partly restored the female reproductive deficiencies observed in *Cyp6g2*^*−/−*^ (Fig. 4). Fourth, the JHB3 and JH III titer decrease significantly in *Cyp6g2*^*−/−*^ larvae (Fig. 5). Fifth, in *D. melanogaster* Kc cells, overexpression of *Cyp6g2* and *jhamt* converted FA into JHB3, overexpression of *Cyp6g2* converted MF into JHB3 and JH III respectively. Overall, our studies presented compelling evidence supporting the role of Cyp6g2 as the major JH epoxidase in JH biosynthesis in *D. melanogaster*, mainly for FA and minorly for MF. However, further investigations are required to elucidate the specific enzymatic properties of Cyp6g2, including its substrates and products. Unlike CYP15 enzymes, Cyp6g2 may not have such a strong substrate specificity, as its overexpression conferred increased tolerance to various insecticides, including the organophosphate insecticide diazinon, the neonicotinoid insecticide nitenpyram, and dichlorodiphenyltrichloroethane (DTT) [27–29, 39]. Considering that epoxidated JH is dispensable for insect embryonic development, it is interesting and requires further research to understand why the embryonic lethality of the *Cyp6g2*^*−/−*^*::jhamt*^*2*^ double mutants was lower than that of the single mutant (Fig. 2C) [19]. Additionally, the embryonic lethality of the *Cyp15c1*^*−/−*^*; jhamt*^*−/−*^ double mutant was also lower than that of the single *jhamt*^*−/−*^ mutant in silkworm [40].

### The order and roles of JH epoxidase and JHAMT during insect evolution

Although JHAMT has been proposed as a rate-limiting enzyme and key enzyme in JH biosynthesis [41–43], the order of the last two steps in JH synthesis has been suggested to depend on the substrate specificity and affinity of the JH epoxidase [44]. In Lepidoptera and higher dipteran species, JH epoxidase exhibited higher affinity for FA, leading to epoxidation preceding methylation, whereas in most other insects, JH epoxidase showed higher affinity for MF, resulting in the esterification of FA to form MF occurred before MF epoxidation [38, 44]. The functional roles of JH epoxidase and JHAMT in development and reproduction have been reported in several insect species, including the domestic silkworm *B. mori*, the red flour beetle *T. castaneum*, the mosquito *A. aegypti*, and the fruit fly *D. melanogaster* (as demonstrated in this study). Each of these insects displayed distinct phenotypes upon knockdown or knockout of JH epoxidase or JHAMT. In *B. mori*, which usually undergoes five larval instars, both genes are functionally important for proper metamorphosis and indispensible for JH biosynthesis [14, 40]. In *T. castaneum*, which typically undergoes 6–8 larval molts, RNAi-mediated knockdown of *JHAMT* in the fourth or fifth instar caused precocious metamorphosis while knockdown of *CYP15A1* did not cause precocious pupation [18, 32]. In *A. aegypti*, which usually undergoes four larval instars, JHAMT also shows more important role than the JH epoxidase [19]. *D. melanogaster* larvae invariably develop in three instars, and neither genetic allatectomy nor deficiency in JH receptor genes, *Met* and *gce* [8, 34, 35], nor simultaneous knockout of *jhamt* and *Cyp6g2*, were able to induce precocious metamorphosis in this insect (Fig. 2). Our studies in *D. melanogaster* show that the developmental and reproductive deficiencies of the *Cyp6g2*^*−/−*^ mutants are more pronounced compared to *jhamt*^*2*^ mutants, which is unique among all the insects studied thus far. At 3 h AIW, *Kr-h1* expression was normal in *jhamt*^*2*^ larvae but was reduced in *Cyp6g2*^*−/−*^ larvae, possibly because of *Kr-h1*-inducing ability of JHB3 acid was stronger than MF and *jhamt*^*2*^ larvae own JHB3 acid. Additionally, our previous results also showed that *Kr-h1* expression was normal in the fat body of *jhamt*^*2*^ larvae [5]. An alternative explanation is that Cyp6g2 plays essential roles in the larval development and ovary maturation that is not related to its function in JH biosynthesis. In comparison with MF, Cyp6g2 prefers using FA as the substrate, and it is the only enzyme identified to epoxide two sites in the JH backbone, thus producing JHB3 as the major JH in higher dipterans (Fig. 6).

Moreover, we found the occurrence of precocious metamorphosis when *jhamt* and *CYP15A1* were depleted by RNAi in the hemimetabolous species, the American cockroach, *P. americana*, which usually shows 14 nymphal instars. And the knockdown of *jhamt* showed a higher proportion of precocious metamorphosis (S. Z. and S. L., unpublished data). Interestingly, in adult *P. americana*, both *jhamt* and *CYP15A1* RNAi depletion equally inhibited vitellogenesis and ovarian maturation [45]. In contrast, in another hemimetabolous insect model, the desert locust *S. gregaria*, the knockdown of *jhamt* resulted in a delay in sexual maturation while the knockdown of *CYP15A1* had no effect [46]. These studies preliminarily showed that the relative importance of JH epoxidase and JHAMT in adult reproductive fitness depends on the order of epoxidation and methylation. However, their functional differences in development also partially depend on unique characteristics of each insect, such as the number of larval molts.

### Other methyltranferase and epoxidases possibly involved in JH biosynthesis in D. melanogaster

In most insect orders, JH III is the only JH homolog produced by the CA. Compared with other insects that exclusively produce JH III, the last two steps of the JH biosynthetic pathway in *D. melanogaster* are much less clear [5]. The only established fact is that FA is the common precursor for JHB3, JH III, and MF in *D. melanogaster* [21]. As the exact biochemical pathway for the production of JH III and JHB3 in *D. melanogaster* has not been fully characterized, there may be two branches within this pathway, one producing the more abundant juvenile hormone JHB3 and the other producing JH III [47]. Our previous studies showed that overexpression and mutation of *jhamt* led to an increase and decrease in JHB3 biosynthesis, respectively, without affecting the production of JH III and MF. This suggested that JHAMT governs JHB3 biosynthesis in the CA [5]. Consistent with previous results, in this study, we observed that the whole-body JHB3 titer decreased by 60–70%, JH III titer decreased by 50% in *jhamt*^*2*^ larvae in comparison with the *w*^*1118*^ larvae. Another recent study also showed that JH III titers were reduced by 50% and JHB3 titers were reduced by > 90% in larval hemolymph of a newly generated *jhamt* mutant line [48]. These results demonstrated that the mutation of *jhamt* does not completely block JH biosynthesis in *D. melanogaster*. The MF titer was dramatically increased in *Cyp6g2*^*−/−*^*::jhamt*^*2*^ double mutants. This implied that the existence of other minor methyltransferases that could convert FA into MF and JHA into JH in the CA of *D. melanogaster*. Similarly, the mutation of *Cyp6g2* led to sharply reduced JHB3 levels, but some JHs still remained. This is in contrast to mosquito *A. aegypti* again, where mutation of JH epoxidase CYP15A1 completely eliminated the production of JH III, causing JH III titer in the hemolymph to completely disappear [19]. Furthermore, the residual JHB3 and JH III levels in *Cyp6g2*^*−/−*^*::jhamt*^*2*^ double mutant larvae were not nil and topical application of FA could partially rescue some *Cyp6g2*^*−/−*^*::jhamt*^*2*^ double mutant larvae into eclosed adults, suggesting the presence of other minor JH epoxidases existed in the JH biosynthesis pathway in *D. melanogaster*. One potential candidate is Cyp6a2, as its orthologous protein Cyp6a1 has been shown to epoxidase MF into JH III [49]. These findings also indicated that JH biosynthesis in mosquitoes is limited to CA, but in *D. melanogaster*, the last steps of JH biosynthesis extend beyond the CA and need to be further investigated.

## Conclusions

Our research collectively established that Cyp6g2 as the major JH epoxidase in the higher Dipteran represented by *D. melanogaster*. The vast majority of FA was epoxidized into JHB3 acid by Cyp6g2 firstly, and then methylated into JHB3 by JHAMT. A small proportion of FA was methylated into MF by JHAMT or other unknown methyltransferase firstly, then epoxidized into JHB3 or JH III by Cyp6g2 or other unknown epoxidase (Fig. 6). These findings laid the groundwork for the further understanding of JH biosynthesis and paved the way for developing specific Cyp6g2 inhibitors as insect growth regulators or insecticides.

## Methods

### Flies and genetics

The putative promoter sequence (2002-bp length: − 2002 to 0 bp, from the translational start site of *Cyp6g2*, the sequence was provided in Additional file 1: Supplementary text) of *Cyp6g2* was amplified as a Sac II-BamH I fragment, and cloned into the pChsGAL4 plasmid to generate the *Cyp6g2-GAL4* construct. The coding sequences of *Cyp6g2* with a C-terminal V5 epitope tag or His epitope tag were cloned into the pUAST vector. Then these constructs were used to produce transgenic flies using P-element-mediated germline transformation by the Core Facility of *Drosophila* Resource and Technology, Center for Excellence in Molecular Cell Science (CEMCS), Chinese Academy of Science (CAS).

*w*^*1118*^, *jhamt*^*2*^, *jhamt-GAL4*, and *JHRR (JH Response Region)-LacZ*, *UAS-GFP* were reported previously [5, 31]. *UAS-Cyp6g2-RNAi*, *UAS-Cyp305a1-RNAi*, and *UAS-Cyp303a1-RNAi* were obtained from the Vienna *Drosophila* RNAi Center. Other flies used in this paper were generated by recombination. All fly strains in this paper were grown at 25 ℃ on standard cornmeal/molasses/agar medium.

### Cell culture and transient transfection

*D. melanogaster* Kc cells were cultured in Schneider’s *Drosophila* medium (21,720,024, Thermo Fisher) supplemented with 5% fetal bovine serum (SH30071.03IH30-45, HyClone). The *pActin-Gal4*, *pUAST-Cyp6g2-V5*, and *pAC-DsRed2-ER* plasmids were transiently transfected as previously described [6, 33].

### Chemicals and topical application of JHs or JH analogs

FA (S-0151), MF (S-0153), JH III (S-0155), and JHB3 (S-0157) were purchased from Echelon Bioscience. Methoprene (S185858) was purchased from Shanghai Aladdin Biochemical Technology Co. Ltd. All JHs or JH analogs used for topical application were dissolved in acetone at a concentration of 6 μg/μl. Third instar larvae (L3D1) were topically treated with 0.5 μl of a variety of concentrations (0–6 μg/μl) of JH mimics diluted in acetone, three biological replicates (each replicate involved 50 L3D1 larvae) were performed. Other details were described in our previous published papers [5, 8].

### Quantitative real-time PCR

Total RNA was extracted using AG RNAex Pro Reagent (Accurate Biotechnology, Hunan, China) according to the manufacturer’s instructions. Then 2 μg of total RNA was reversely transcribed into cDNA using HiScript III RT SuperMix for qPCR (+ gDNA wiper) (Vazyme Biotechnology, Nanjing, China). Quantitative real-time PCR (qRT-PCR) was performed using Hieff® qPCR SYBR Green Master Mix (Low Rox Plus) (Yeasen Biotechnology, Shanghai, China) and the Applied Biosystem™ QuantStudio™ 6 Flex Real-Time PCR System. The primers used for qRT-PCR are listed in Table S1 (Additional file 1). Three or more biological replicates (each replicate involved 20 or more individuals) were performed as previously described [6, 33].

### Sesquiterpenoid extraction and measurement

Groups of 100 larvae were homogenized in 400 μL ice-cold acetonitrile and were processed by the acetonitrile/pentane extraction method described previously [50]. The recovered organic fraction was dried under N_2_ and stored at − 20 ℃ until used. Sesquiterpenoid titers were determined using the recently developed LC–MS/MS protocol by the Center of Pharmaceutical Technology, Tsinghua University [51].

### Enzyme assays of Cyp6g2, JHAMT, and DpCYP15A1 in Kc cells

Overexpression of *Cyp6g2*, *JHAMT*, and *DpCYP15A1* in Kc cells was achieved using a GAL4/UAS system by co-transfecting with *pActin-Gal4* construct. *UAS-EGFP* was used as a negative control. Twenty-four hours after transfection of Kc cells in 100-mm dish. The old medium was replaced with 15 ml of fresh medium. FA or MF was (100 μM at final concentration) added to the medium. After incubation another 24 h, cells were collected by centrifuging for 5 min at 1000 × *g*. Then these cells were used for extracting sesquiterpenoids.

### Immunofluorescence staining and microscopy

Brain-RG complexes were dissected from larvae of corresponding time points and stained with antibodies according to standard procedures. The primary antibodies used include LacZ mouse monoclonal antibody (40-1a, Developmental Studies Hybridoma Bank, DSHB), DsRed2 mouse monoclonal antibody (sc-101526, Santa Cruz), V5-Tag (D3H8Q) rabbit monoclonal antibody (#13202S, Cell Signaling Technology), JHAMT rabbit polyclonal antibody (reported previously in [9]), and Cyp6g2 rabbit polyclonal antibody. Secondary antibodies used are Alexa Fluor™ 488 goat anti-rabbit IgG (A-11008; Thermo Fisher), Alexa Fluo™ 594 goat anti-mouse IgG (A-11005; Thermo Fisher). DAPI was used for nuclei labeling. The resulting fluorescence signals were examined under a confocal microscope Olympus FV3000 as previously described [9, 31].

Cyp6g2 rabbit polyclonal antibody was generated by ABclonal Biotechnology Co. Ltd (Wuhan, China). The partial coding sequence of *Cyp6g2* (125aa-300aa) was cloned into *pET28a-SUMO* vector. The recombinant protein was purified from inclusion body with NTA-Ni^2+^ beads. Purified protein was quantified and then used to immunize rabbits.

### Ovary size measurement and fecundity analysis

The ovary in the virgin *Drosophila* on day 7 after eclosion were dissected and photographed with a Nikon SMZ25 stereomicroscope. Then the areas of at least 10 pairs of ovary were calculated. To analyze fly fecundity, 2 tested virgin females were crossed with 3 wild-type males in a new vial of food with a few grains of dry yeast. Flies were transferred every day, and the number of eggs laid was counted every day. For each genotype, six parallel replicates were set. Other details were described in our previous published papers [5, 31].

### Statistical analysis of data

All of the data were presented as the mean ± standard deviation of three or more independent experiments and were analyzed using Student’s *t* test and ANOVA. Asterisks indicate a significant difference as calculated using the two-tailed unpaired Student’s *t* test (*, *p* < 0.05; **, *p* < 0.01; ***, *p* < 0.001). For ANOVA, the bars labeled with different lowercase letters are significantly different (*p* < 0.05).

### Supplementary Information

Additional file 1. Figure S1. The changes in JH-associated phenotypes upon CA-specific knockdown of Cyp303a1, Cyp305a1 and Cyp6g2. Table S1. Primers used for qRT-PCR in this study. Supplementary text. The promoter sequence of Cyp6g2 used for generating Cyp6g2-Gal4 transgenic flies.

Additional file 2. The individual data values for Fig. 1A, C,  Fig. 2C, D, G, Fig. 3D, Fig. 4A, C, C’, Fig. 5A-C and Fig. S1A-C.

## Data Availability

All data generated or analyzed during this study are included in this published article and its supplementary information files. The datasets used and/or analyzed during the current study are available from the corresponding author on reasonable request.

## Abbreviations

FA: Farnesoic acid
MF: Methyl farnesoate
JH III: Juvenile hormone III
JHB3: Juvenile hormone III bisepoxide
JHA: Juvenile hormone acid
CA: Corpora allata
RNAi: RNA interference
qRT-PCR: quantitative Real-time PCR
JHAMT: Juvenile hormone acid methyltransferase
DpCYP15A1: *Diploptera punctate* CYP15A1
Met: Methoprene tolerant
Gce: Germ cell-expressed bHLH-PAS
Kr-h1: Krüppel homolog 1
yp: Yolk protein
LC–MS/MS: Liquid chromatography-tandem mass spectrometry
AIW: After initiation of wandering stage
EW: Early wandering stage
WPP: White prepupal stage
APF: After puparium formation
Br-RG: Brain-ring gland complex
