# Supplementary material for: Cyp6g2 is the major P450 epoxidase responsible for juvenile hormone biosynthesis in Drosophila melanogaster
Source: BMC Biol. 2024 May 13;22:111. doi: 10.1186/s12915-024-01910-4 (PMC11092216; doi:10.1186/s12915-024-01910-4)
Supplement: Supplementary file 1 — Additional file 1. Figure S1. The changes in JH-associated phenotypes upon CA-specific knockdown of Cyp303a1, Cyp305a1 and Cyp6g2. Table S1. Primers used for qRT-PCR in this study. Supplementary text. The promoter sequence of Cyp6g2 used for generating Cyp6g2-Gal4 transgenic flies. [file 12915_2024_1910_MOESM1_ESM.docx]

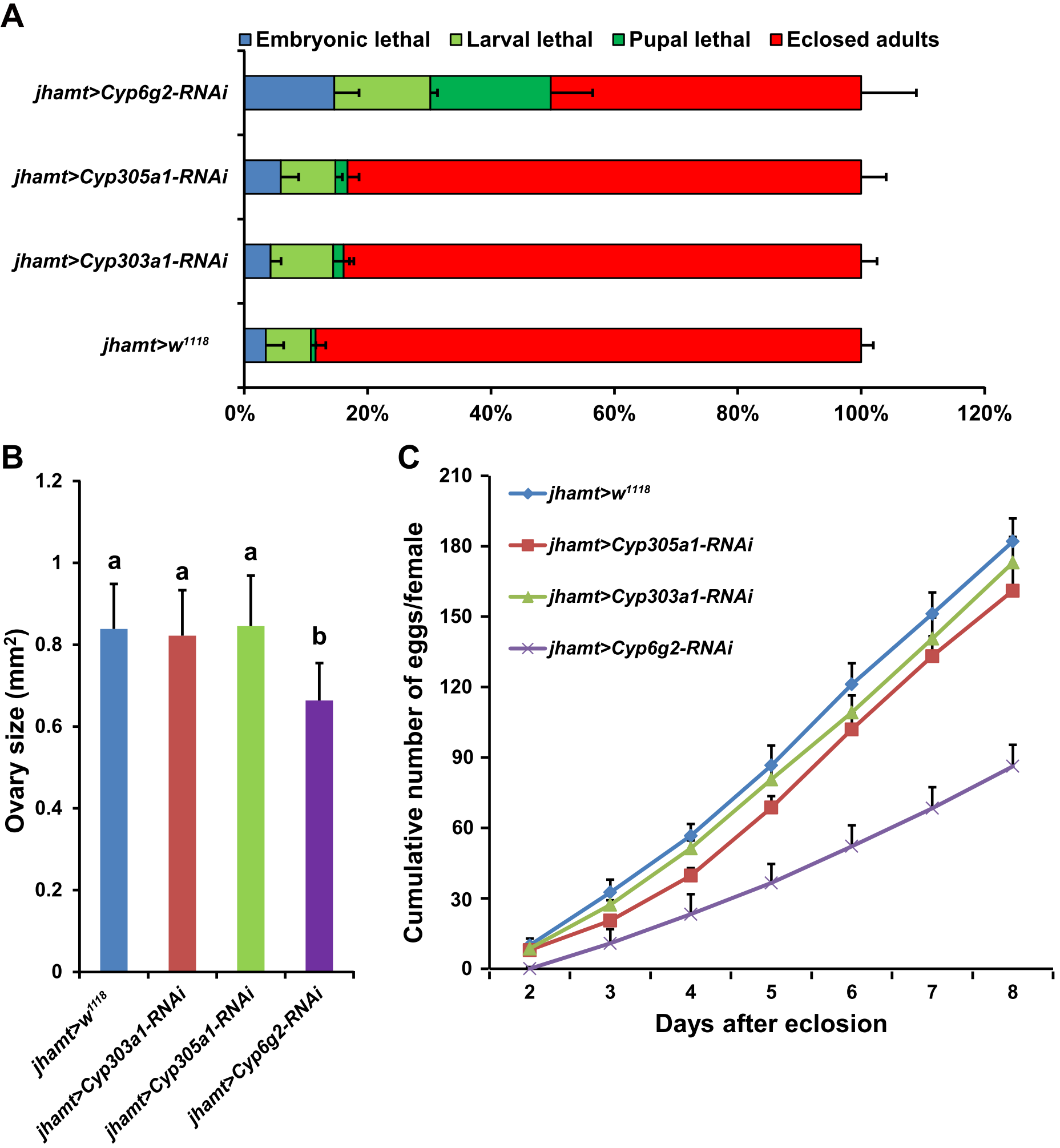


**Additional file 1: Figure S1. The changes in JH-associated phenotypes upon CA-specific knockdown of *Cyp303a1*, *Cyp305a1* and *Cyp6g2*.**

(A) Developmental phenotype of CA-specific knockdown of *Cyp303a1*, *Cyp305a1* and *Cyp6g2* (*n*=3).

(B) Changes in ovary size upon CA-specific knockdown of *Cyp303a1*, *Cyp305a1* and *Cyp6g2* (*n*=4).

(C) Changes in egg production upon CA-specific knockdown of *Cyp303a1*, *Cyp305a1* and *Cyp6g2* (*n*=3).

**Additional file1: Table S1. Primers used for qRT-PCR in this study.**

| Gene name | Forward (5’-3’) | Reverse (5’-3’) |
| --- | --- | --- |
| *Cyp6g2* | GGTACTGCTGATCCTCGTGGCA | CGGGACTCATTCGCATGTTGA |
| *jhamt* | CCGATGGAGAGGATGCACTC | AGAACCTGGAATCTGGTGCG |
| *Kr-h1-α* | AAGTGGTGGTCGTTAAGTGTTAT | TGTTATGTCGTATTCGGGCTAT |
| *Kr-h1-β* | AAGTGTTGTTTGTACGGGTGTT | GATGATTGCGGTATTGGTGG |
| *yp1* | TCCAAGCTGAACACCTATGAGCGT | TGGTATCGAAGGGCATGTCCAACT |
| *yp2* | AGGAAGCAGAACGGTGAACAGGAT | TACTGCTCAAAGTCCTCGATGGCA |
| *yp3* | TACCACGTTGGCCAAATCAAGCAC | TGGCCGTCTCCACATAGTTGTTCA |
| *rp49* | GACAGTATCTGATGCCCAACA | CTTCTTGGAGGAGACGCCGT |

**Additional file 1: Supplementary text. The promoter sequence of *Cyp6g2* used for generating *Cyp6g2-Gal4* transgenic flies:**

TTCGTTTTAAGTTCAAATTTTAGGGAGCTATTTAGATTATCTATTCTATTTAGATTATAGGAACAAAGGTGATTACATATCACAGCCGATTGTTTTAGGATGCCTATAACTCTACAAACTACTCCTGCAGAATCTTAGGTATATCTCATTCTCAGACTCCAACATGAAGGACTTTGGATTAAAGCGAATCTCCGATACTGTTCGCTCGCAAGTCTCAACCCAATATTGCTTTAAAATGTGCACTATGCCCAGTTTCAATTGGAGTACGCCCAGGCGACTACCAATACAGCCATGTGGACCTGCTCCAAAGGGTATGTAGGTCATCGGGTGGATGTGCCTAGATCGCTCCGGACCGAATCTCTCGGGATCAAAGACGCAGGGCTCCGGCCAAAACTGGAATTTAGATTGACATAAGTAAGTTGGGATAATCATCACCTTTGGTTTTAAATCTAACCCTTTCATCGCGATGGAGGCCGAGTATTGAGATGTATGCTGGCATCCCTGGTGGCACTATGAAATCCACATGCGGTTGCAGTGAGAATCCCTCGGATGCCGAACTGGTGCACTCTCTGTTGACGAAAGCAGCAGCCGGATAGAGACGCAGTGCCTCCAAACACACCATTTTGAGATAGGGCAAAGTCATCAGTGTGTCATAGCTTAGGGTAGCAGTGGAGATGAAGGCTTCTCGTAGCTCACTCCTCAGTCGTTCTTGAATATCCGGAGCCTTGGCCAACTCATAGAGAGTGAATCCCATCAGGGCTGAGGAGGTTTCAAATCCGGCCAGCAGTATGATACCCGCCTGCGAAGCAACAAAGTCCGGATGCTGGGAATAGTGGTTGGATGAGCGGCTCAACTGAAAGTGCTGCAATTGATTGATGAGATCTCCTTTGGTGGGCTCGTGATGATCATCTACCAAGTGCCTCATATAGCGCGCATAGTCTTCGGTAAAAACTTTGGGCTTCAGCACACCCGTCCACTTGGGCAGAAAGAATACACTCATGAAGTCCAAAACCTTGCGGGGATTGGTGTTAAATAGCTCCTTGGTTTTTGTTATCAACTCGGATCGACCACGACGCAATCCGCCGACATTCAGGCTGTAGAAGAGATTCCCCGTCACGTCGGTGGTGTACAACTGGCACATTCTTCCCAACGGGAGAACGCGCTCCAGCCGATCCCCCAGCTTCCTGTTGAGGTACTGCTCCAAGTCGCTGGCCACATCCAACATTTGGCTGTACATGACATCCCGCATACGGCCGCTGGTGAAGAGCTGGGACATGCATTGCCGACTCTCCTTCCAGTGATGGTACTTAGCCAGCGGTAGCGTCAGTGCACCCATGGGATCTCCGGCGTCCGCCGATTCGAACCGGTTGAGAAAGTTGTTGAAATTCTTAATCAACACCTGGCGGATTAGCTCCGGATCCCGAACCATAAGCGCCGGCGTTTGGAATATGAAGAAGCCAACGATCTTCGCCTGACCATTTCGTGGATCTGCGTAGAGCTGCCGAAAGAGATCGCCAAAAGAAATGCGCAGAAATAGAAGTTGTCCCAAATTGCCCATTGGTGACCAAGAGGAGGGTGGCAGGTGCGGAATCCCACGGCTCCTGAAGTAATCGTACTTGTGCCTTAACCAAAAATTCAGAGTCACAATGGTGAGCAACAGCAGCCAGATGAGCAGCATGGTGTCTATAAGATATAAGATAGAATATTATAACTATGAAATATATACCAGCATTAGGTAAAGATAGCTAAGATCCAAAAAGTCGTTCTTTTGAATGAGGAGGAGACCTTGTTCACGACCGTCAAGAAATGCATTTCCATATGCATAAGTTGAACCTGACCCACTTAACATCTCGACAAGCACTTCCATTCACAAATAGATTCATTCATTGCACTCAGGATCATCTTGATGATCTCATAGTAGAGTTTCCGCTTACCCAAAGCTTTAACTCTATGGTGCTGGCAATATTTCAGCCAGTTGGACATTGGGTTTGGTGGCGTTAGC
